# Completely calcified mastic kidney

**DOI:** 10.11604/pamj.2023.46.34.39574

**Published:** 2023-09-25

**Authors:** Hicham El Bote, Jihad Lakssir

**Affiliations:** 1 Sultan Moulay Slimane University, Department of Anatomy, Faculty of Medicine and Pharmacy, Beni-Mellal, Morocco,; 2 Regional Hospital of Beni Mellal, Department of Surgery, Beni-Mellal, Morocco,; 3 University of Rabat, Department of urology A, Ibn Sina Hospital, Rabat, Morocco

**Keywords:** Mastic kidney, calcified kidney, renal tuberculosis

## Image in medicine

A 64-year-old man who had a history of pulmonary tuberculosis (TB) with extrapulmonary relapse at the urogenital system that was treated and declared cured 20 years ago, presented a simple left lumbago. Ultrasonography detected a large right kidney that looked destroyed with a strong acoustic shadow, the left kidney was normal, non-contrast computed tomography showed a right kidney with a rounded calcification involving the entire parenchyma which was damaged. Although the QuantiFERON-TB Gold test was positive, the Löwenstein-Jensen culture and a polymerase chain reaction (PCR) test in urine were negative as well as the chest X-ray. Thus, the diagnosis of mastic kidney complicating previous renal tuberculosis was made. The collegial decision with the nephrologists was a therapeutic abstention due to normal renal function and the absence of symptoms. According to the latest World Health Organization (WHO) report, TB is the second most common cause of death from a single infectious agent after coronavirus (COVID-19). The disease usually affects the lungs (pulmonary TB), but can also affect other parts of the body (extrapulmonary TB). Renal TB is the most common form of urogenital TB. It accounts for 20% of extrapulmonary localisations and 4-8% of patients with pulmonary TB develop destructive lesions in the genitourinary tract. The primary infection in the kidney remains confined to the glomeruli, but on reactivation, *Mycobacterium tuberculosis* spreads to the medulla and interstitium. In the active phase the disease progresses from periodic ruptures, granuloma formation, caseous necrosis and cavitation in the renal parenchyma, while in the cicatricial phase, the host's healing response induces fibrosis, calcium deposition and stricture formation. Mastic or Putty kidney is the radiological appearance of a dystrophic calcification of the unfunctional kidney caused by tuberculosis (auto-nephrectomy).

**Figure 1:**
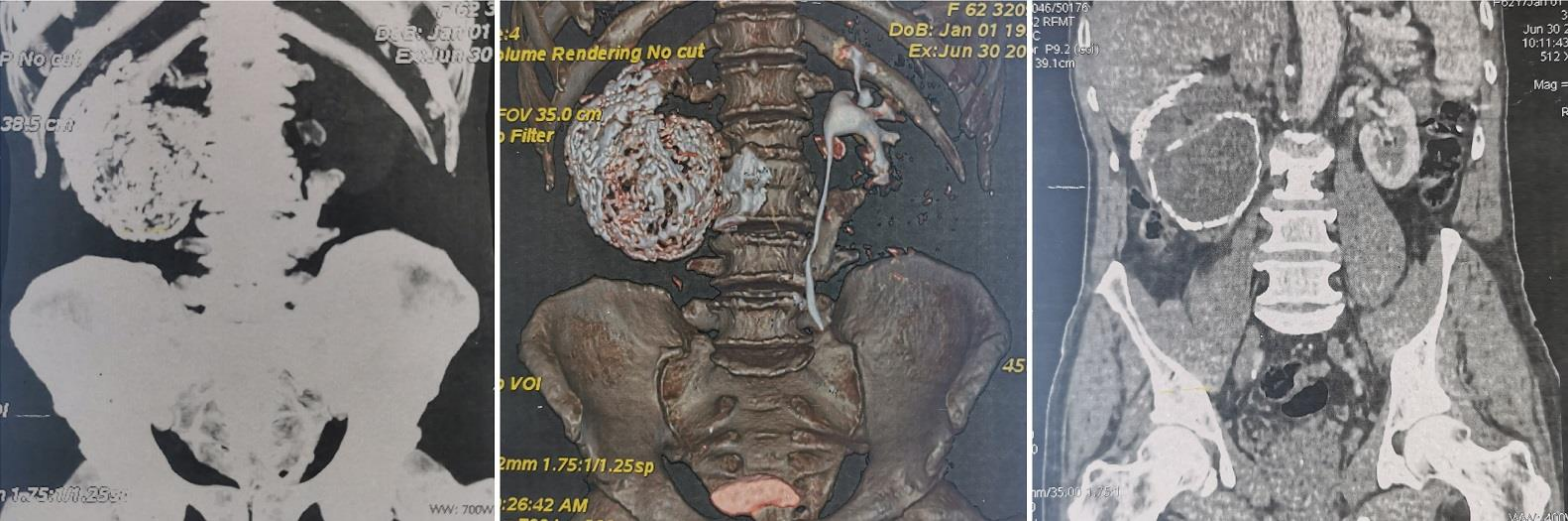
computerized tomography scan sections showing a completely right calcified mastic kidney

